# Supplementary figures and images for: Bipartite binding interface recruiting HP1 to chromosomal passenger complex at inner centromeres
Source: J Cell Biol. 2024 May 23;223(9):e202312021. doi: 10.1083/jcb.202312021 (PMC11116813; doi:10.1083/jcb.202312021)

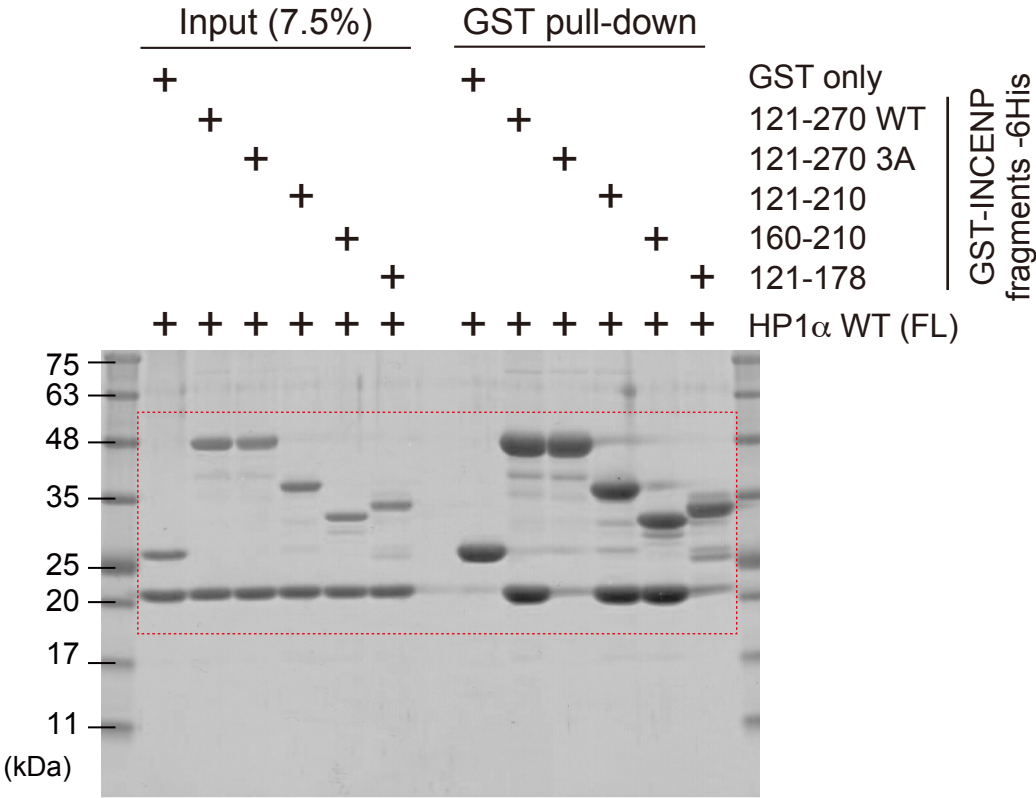

Supplement: SourceData F2 — is the source file for Fig. 2. [file JCB_202312021_SourceDataF2.pdf]

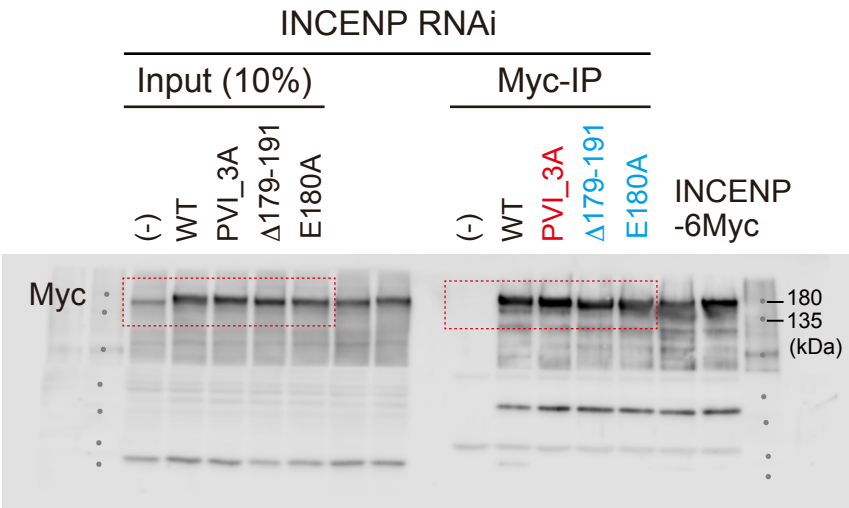

Image 1 (short exposure)

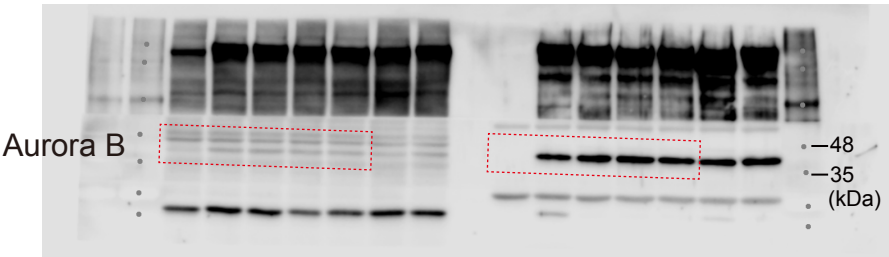

Image 1 (middle exposure)

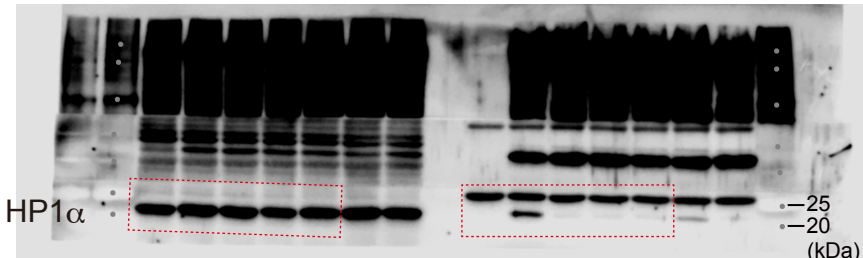

Image 1 (long exposure)

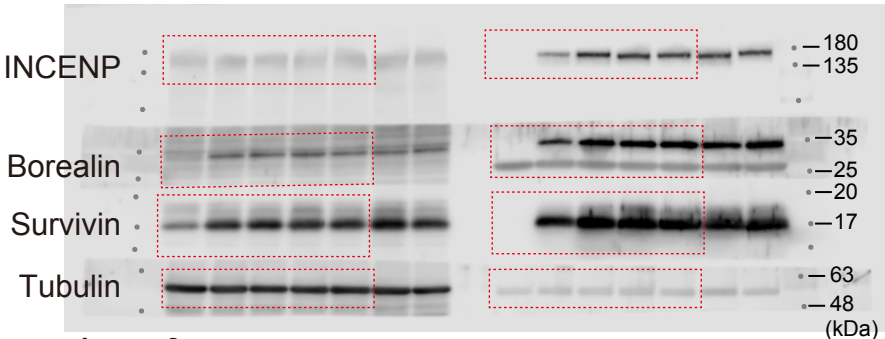

Image 2

Supplement: SourceData F5 — is the source file for Fig. 5. [file JCB_202312021_SourceDataF5.pdf]

Fig. S1 A

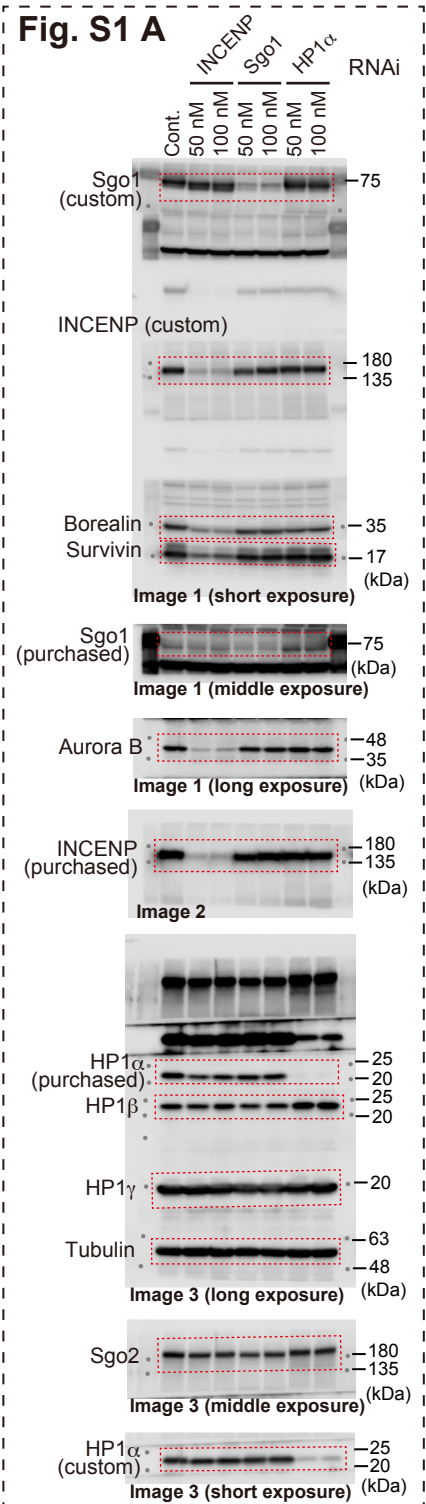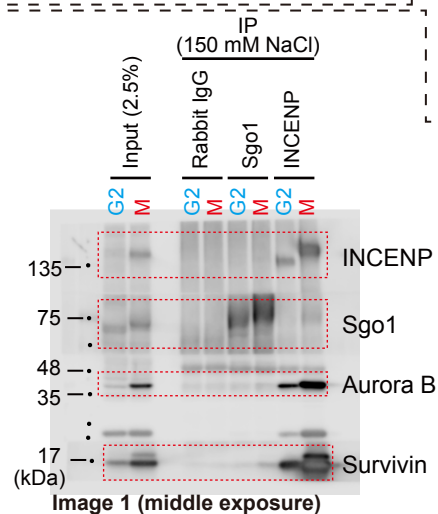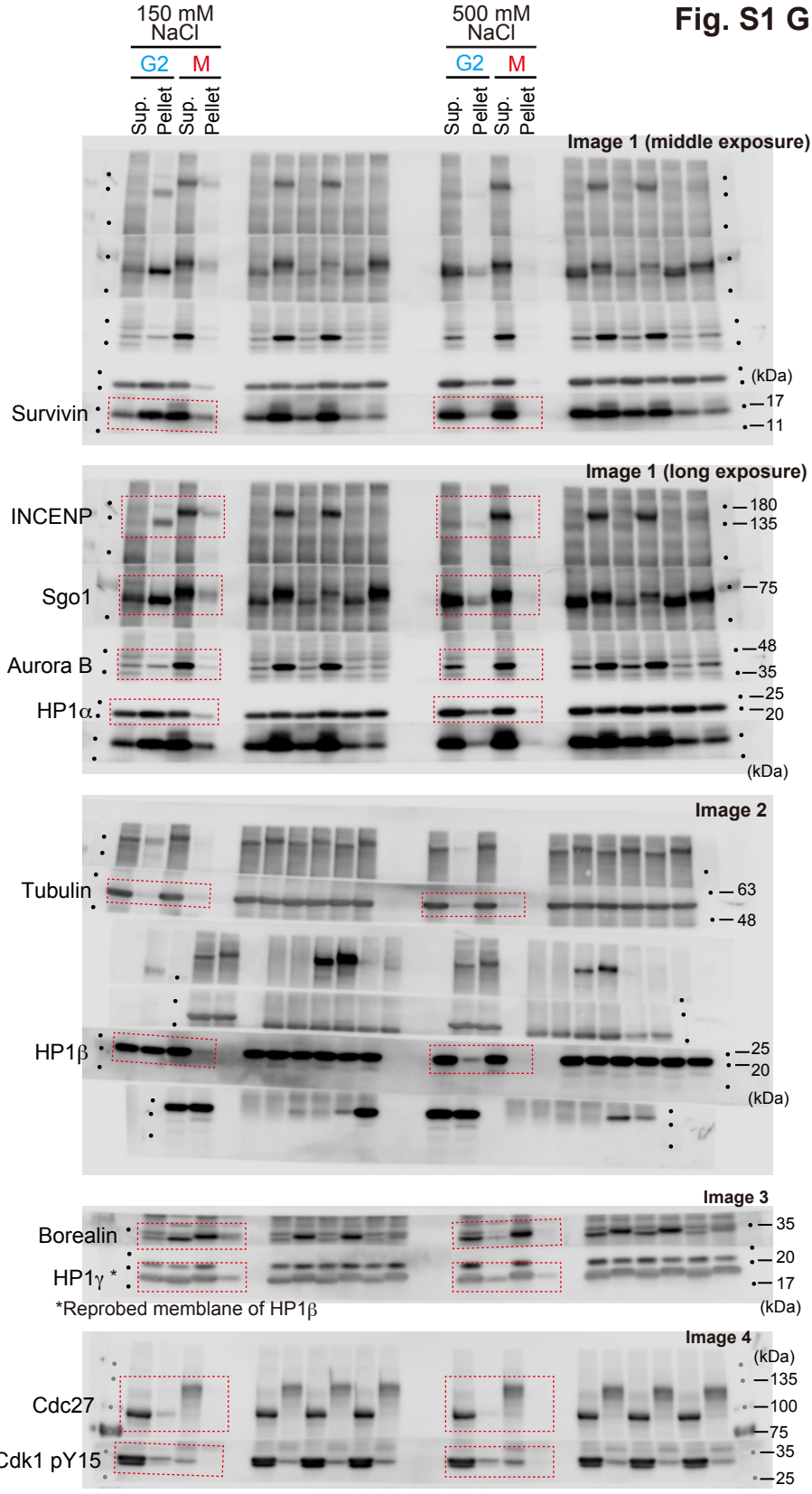

Fig. S1 H

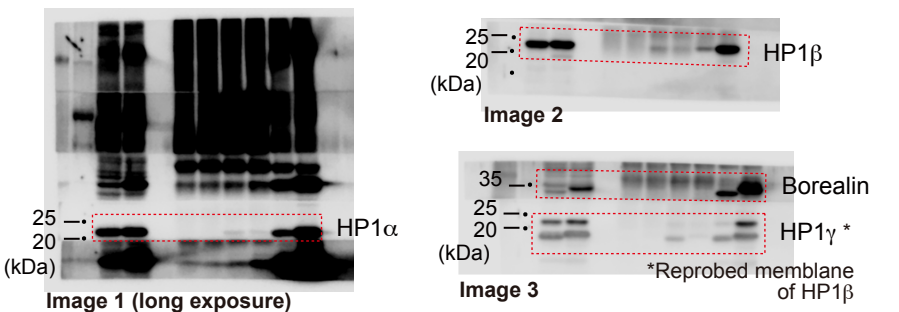

Supplement: SourceData FS1 — is the source file for Fig. S1. [file JCB_202312021_SourceDataFS1.pdf]

Fig. S2 C

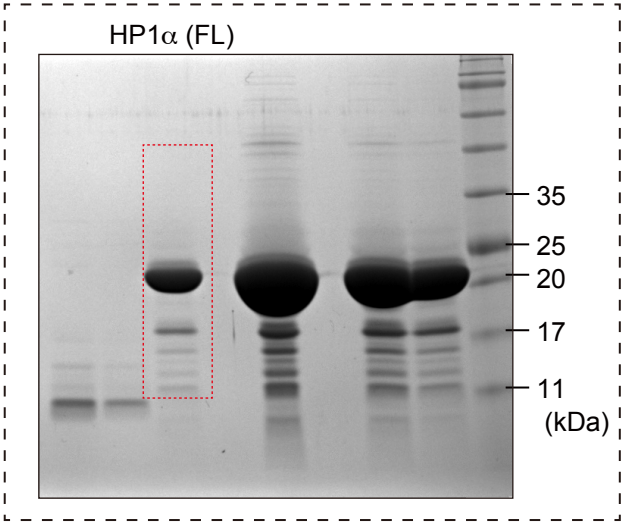

Fig. S2 D

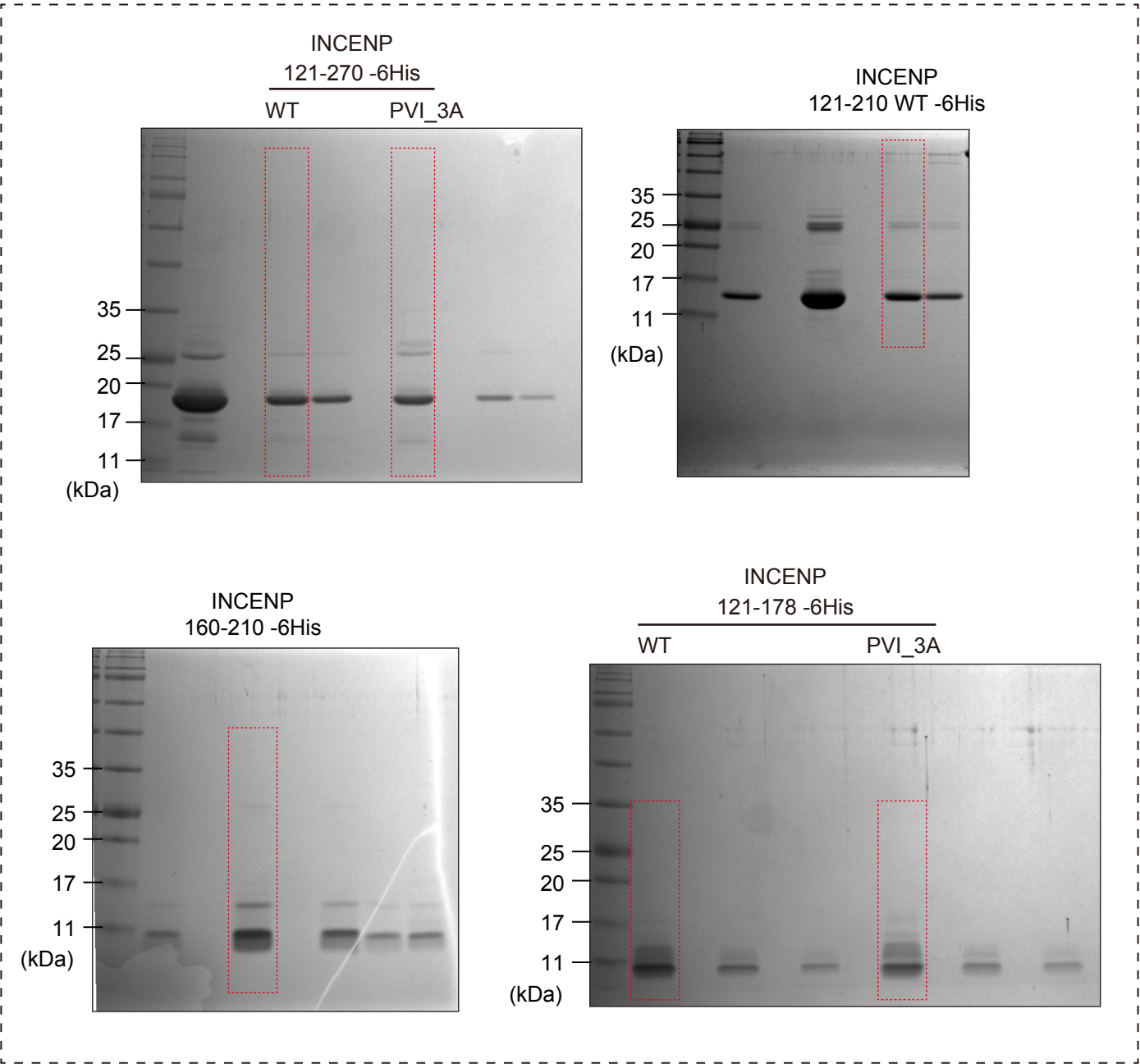

Supplement: SourceData FS2 — is the source file for Fig. S2. [file JCB_202312021_SourceDataFS2.pdf]

**Fig. S3A**

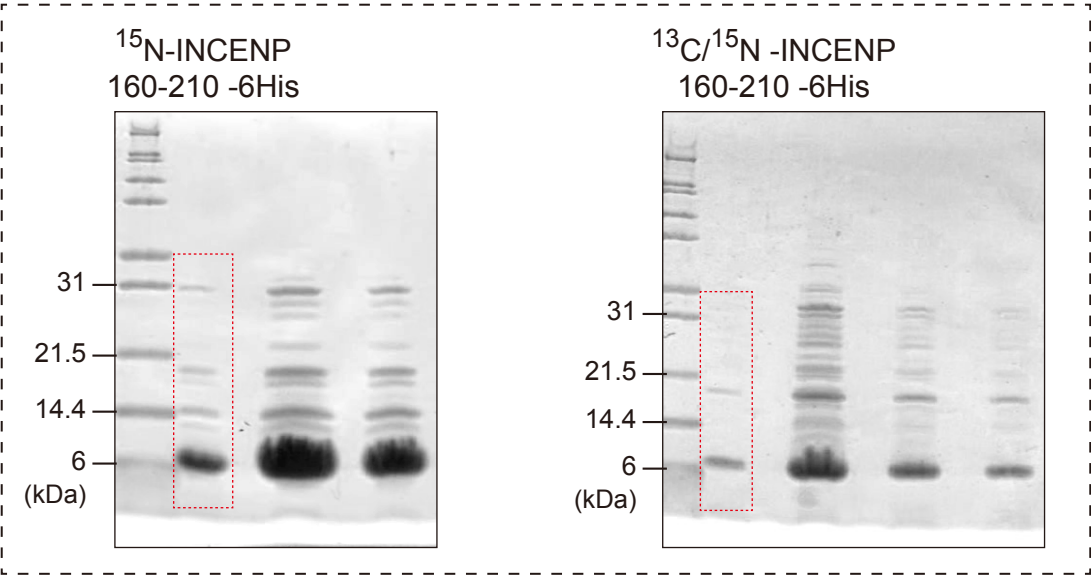

**Fig. S3 B**

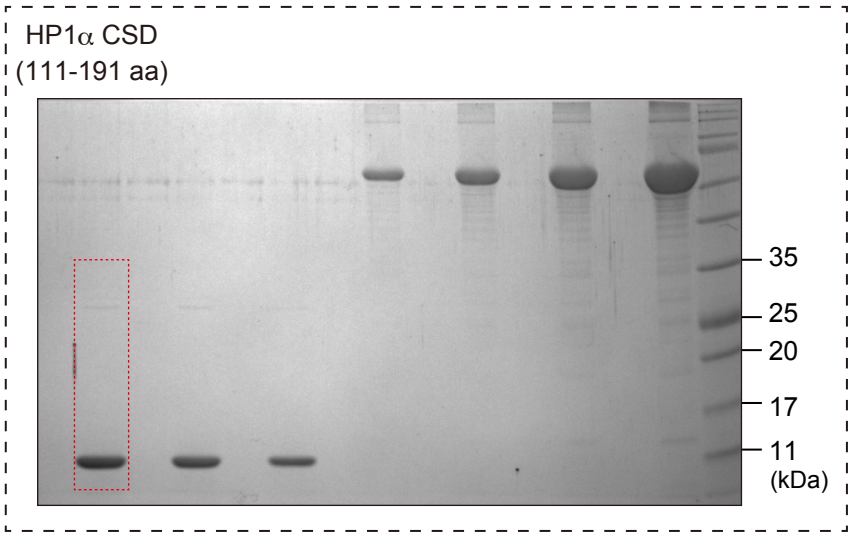

**Fig. S3 C**

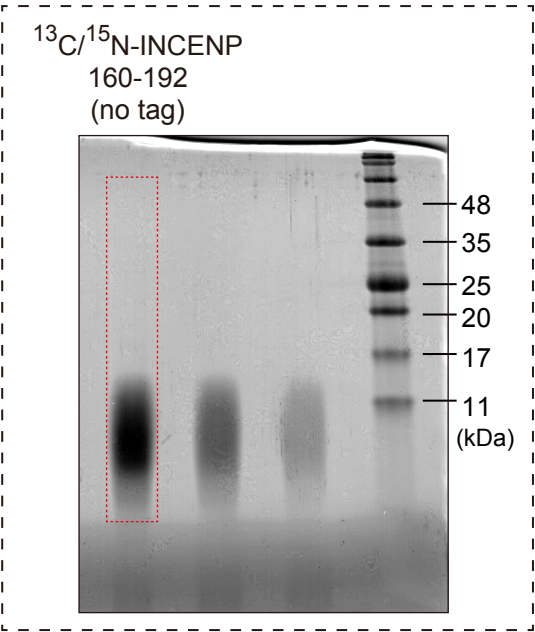

Supplement: SourceData FS3 — is the source file for Fig. S3. [file JCB_202312021_SourceDataFS3.pdf]

**Fig. S4 A**

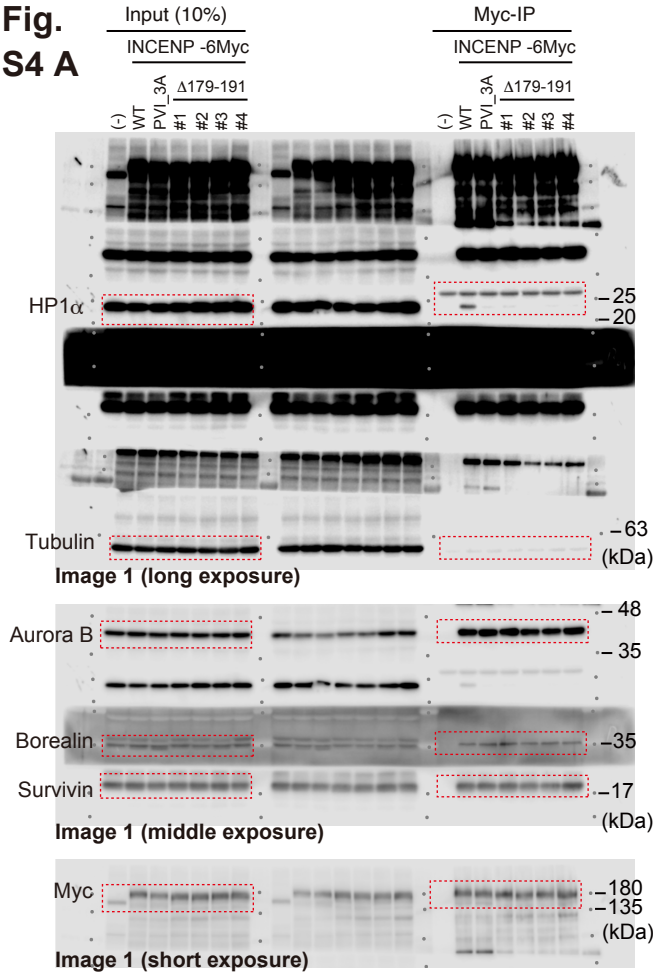

**Fig. S4 B**

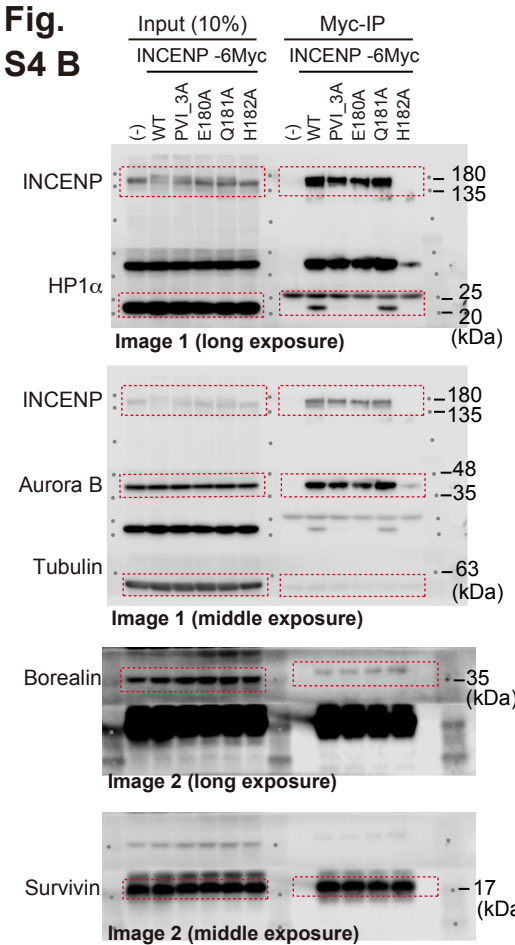

**Fig. S4 E**

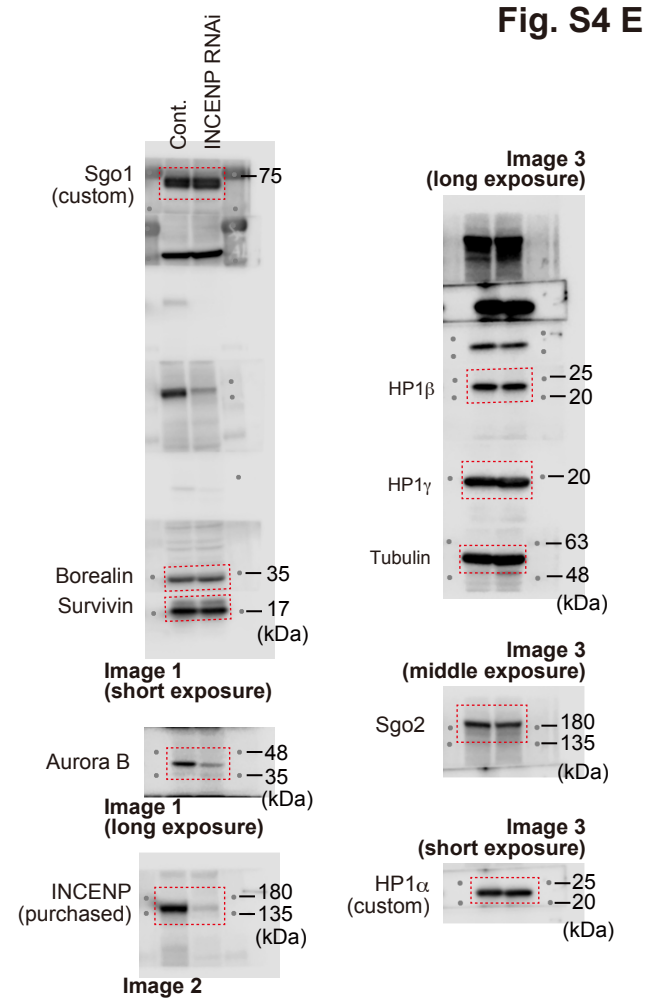

**Fig. S4 F**

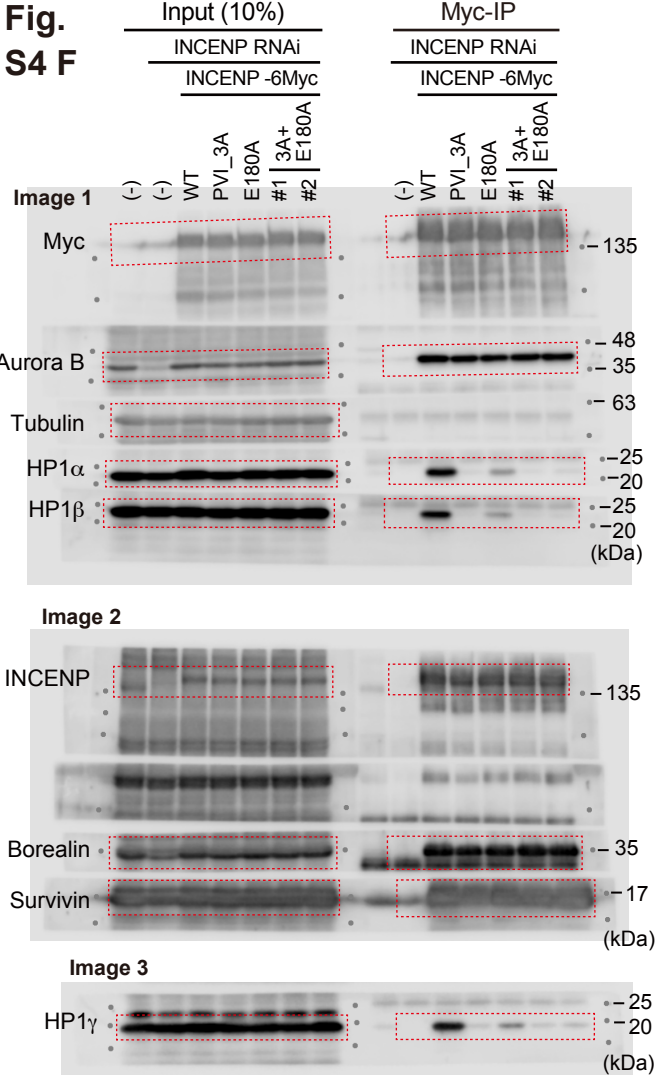

Supplement: SourceData FS4 — is the source file for Fig. S4. [file JCB_202312021_SourceDataFS4.pdf]
