# Supplementary material for: Bipartite binding interface recruiting HP1 to chromosomal passenger complex at inner centromeres
Source: J Cell Biol. 2024 May 23;223(9):e202312021. doi: 10.1083/jcb.202312021 (PMC11116813; doi:10.1083/jcb.202312021)

SourceDataFS5

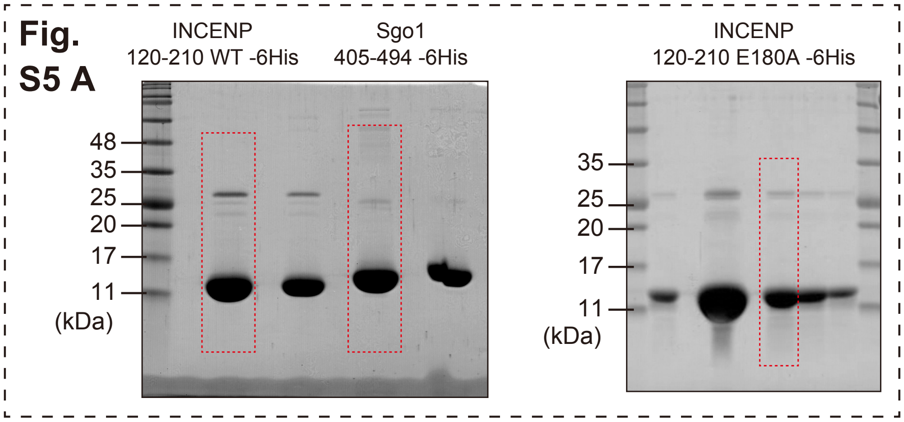

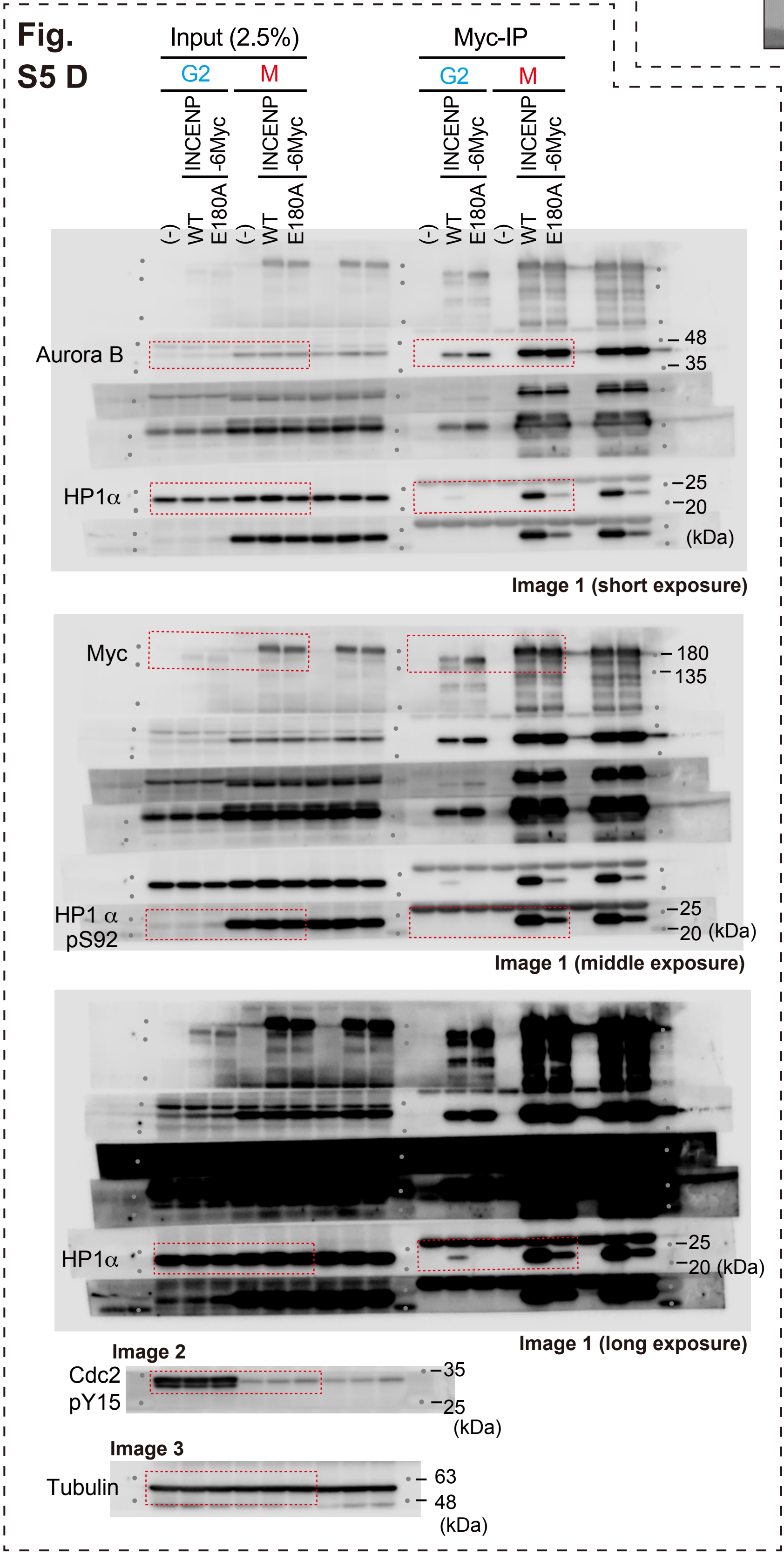

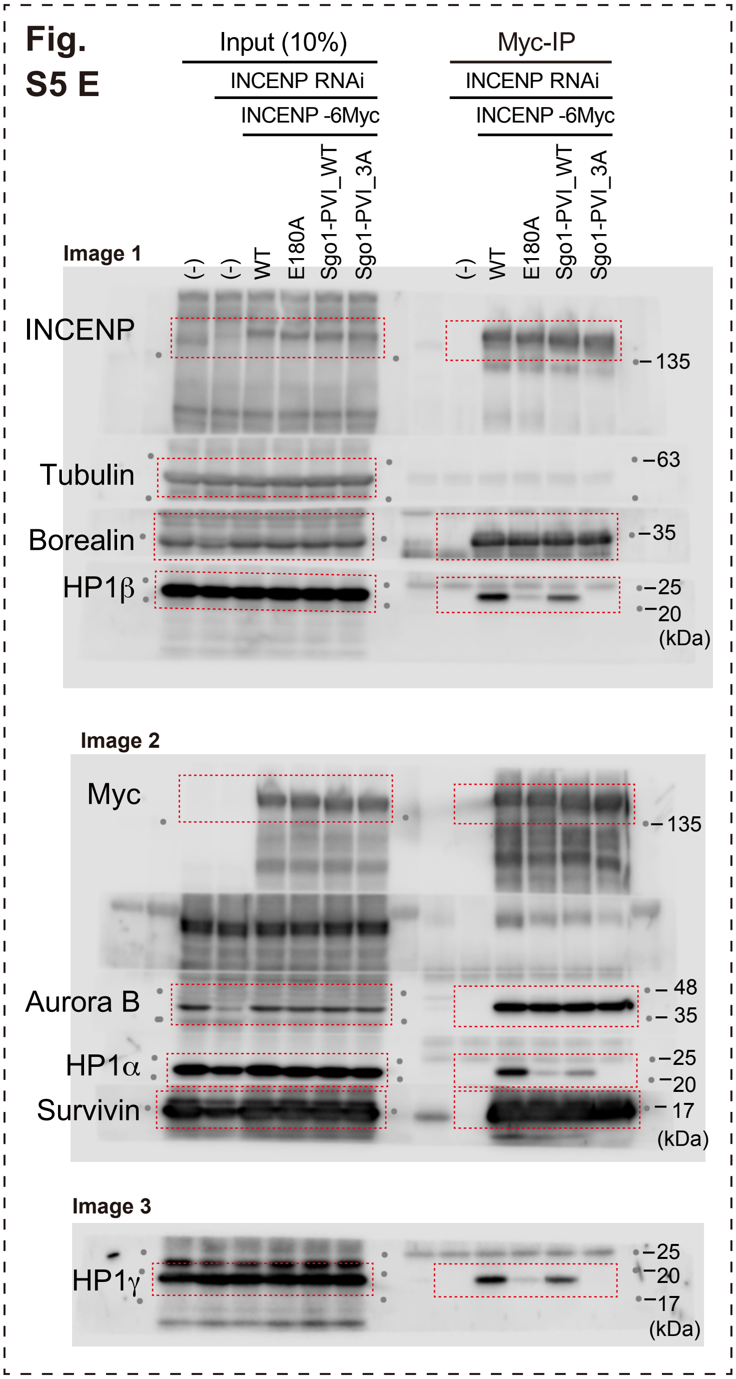

Supplement: SourceData FS5 — is the source file for Fig. S5. [file JCB_202312021_SourceDataFS5.pdf]
